# Mendelian randomization in blood metabolites identifies triglycerides and fatty acids saturation level as associated traits linked to pancreatitis risk

**DOI:** 10.3389/fnut.2022.1021942

**Published:** 2022-10-10

**Authors:** Jiarui Mi, Zhengye Liu, Lingjuan Jiang, Meizi Li, Xia Wu, Nan Zhao, Ziqi Wan, Xiaoyin Bai, Yunlu Feng

**Affiliations:** ^1^Department of Gastroenterology, Peking Union Medical College Hospital, Peking Union Medical College and Chinese Academy of Medical Sciences, Beijing, China; ^2^Master Programme of Biomedicine, Karolinska Institutet, Stockholm, Sweden; ^3^School of Clinical Medicine, Zhejiang University, Hangzhou, China; ^4^Medical Research Center, Peking Union Medical College Hospital, Chinese Academy of Medical Science and Peking Union Medical College, Beijing, China; ^5^Department of Medicine, Tufts Medical Center, Boston, MA, United States; ^6^Department of Clinical Medicine, Peking Union Medical College and Chinese Academy of Medical Sciences, Beijing, China

**Keywords:** Mendelian randomization, metabolites, triglycerides, fatty acids, pancreatitis

## Abstract

**Background:**

There is very limited evidence on the causal effects of blood metabolites on pancreatitis risks. To reveal the causal associations between plasma metabolites and pancreatitis risks, we performed two-sample Mendelian randomization (MR) and Bayesian model averaging (MR-BMA) analyses in European ancestry.

**Methods:**

The summary-level statistics from two genome-wide association studies with 249 and 123 metabolic traits derived from two separate cohorts involving ~115,000 (UK Biobank) and ~25,000 individuals from European ancestry were used for the analyses. The summary statistics of four pancreatitis datasets from FinnGen R5 and two pancreatitis datasets from UK Biobank were exploited as the outcome. We first performed univariable MR analysis with different metabolic GWAS data on multiple pancreatitis datasets to demonstrate the association pattern among different metabolites categories. Next, we exploited the MR-BMA method to pinpoint the dominating factors on the increased risk of pancreatitis.

**Results:**

In the primary analysis with 249 traits, we found that plasma triglycerides were positively associated with pancreatitis risk. Intriguingly, a large number of traits associated with saturation or unsaturation of fatty acids also demonstrated causal associations. The replication study analyzing 123 metabolic traits suggested that bisallylic groups levels and omega-3 fatty acids were inversely correlated with pancreatitis risk. MR-BMA analyses indicated that the ratio of triglycerides to total lipid in various HDL particles played leading roles in pancreatitis susceptibility. In addition, the degree of unsaturation, the ratio of polyunsaturated fatty acids to monounsaturated fatty acids and the level of monounsaturated fatty acids showed causal associations with either decreased or increased pancreatitis susceptibility.

**Conclusions:**

Our MR study provided an atlas of causal associations of genetically predicted blood metabolites on pancreatitis, and offered genetic insights showing intervention in triglycerides and the supplementation of unsaturated fatty acids are potential strategies in the primary prevention of pancreatitis.

## Introduction

Pancreatitis is a common disease featured by autodigestion of the pancreas (1). Acute pancreatitis can transit into recurrent episodes, and further progress into chronic pancreatitis (1). Several modifiable risk factors, including smoking, alcohol consumption, abdominal adiposity and obesity, gallstone diseases and elevated plasma triglycerides levels, have been extensively studied in various cohort studies (2, 3). However, the cause of pancreatitis and what and how various risk factors lead to the increase of risk of pancreatitis are still elusive.

In addition, genome-wide association studies (GWAS), have also identified various genetic variants in pancreatitis susceptibility (i.e., UK BioBank and FinnGen consortium) (4). These studies, all together, indicated that different pancreatitis are sets of complex disorders with the interplay between environmental and genetic factors. With the advent of high-throughput technologies, now we are able to measure hundreds of circulating metabolites as well as perform genotyping in large-scale populations in parallel (5). People have identified large numbers of single nucleotide polymorphisms (SNPs) that show strong associations with plasma metabolites. Mendelian randomization (MR) is an epidemiological method to investigate the causal effects between exposures and outcomes by making use of summary-level statistics of GWAS datasets. The rationale of MR is to exploit valid genetic variants as instrumental variables (IVs) proxying exposures of interest and then use these IVs to assess the causal estimates on the outcomes. In causal inferences, the MR method has several advantages over traditional observational studies, as it is less likely biased by confounders and reverse causality. In addition, the MR method is also suitable to investigate deleterious factors and biological measurements, which are hardly manageable in prospective cohorts. In addition, this method can provide novel insights on disease development and potential therapeutic targets for drug repurposing (6). Previous MR studies have revealed that calcium and triglycerides levels showed causal effects on pancreatitis risk, however, a systematic assessment of plasma metabolites on the pancreatitis susceptibility is still lacking (3, 7).

Here, by employing several MR methods together with two sets of large-scale metabolic profiling GWAS datasets, our study, for the first time, provided genetic evidence on hundreds of blood metabolites and identified several leading factors that were causally associated with pancreatitis risks. Among them, we discovered that the level of triglycerides and the fatty acid unsaturation levels are the leading nutritional risks of both acute and chronic pancreatitis, which indicates that nutritional intervention is a good strategy in the primary prevention of pancreatitis.

## Methods

### Study design

**Figure 1:**
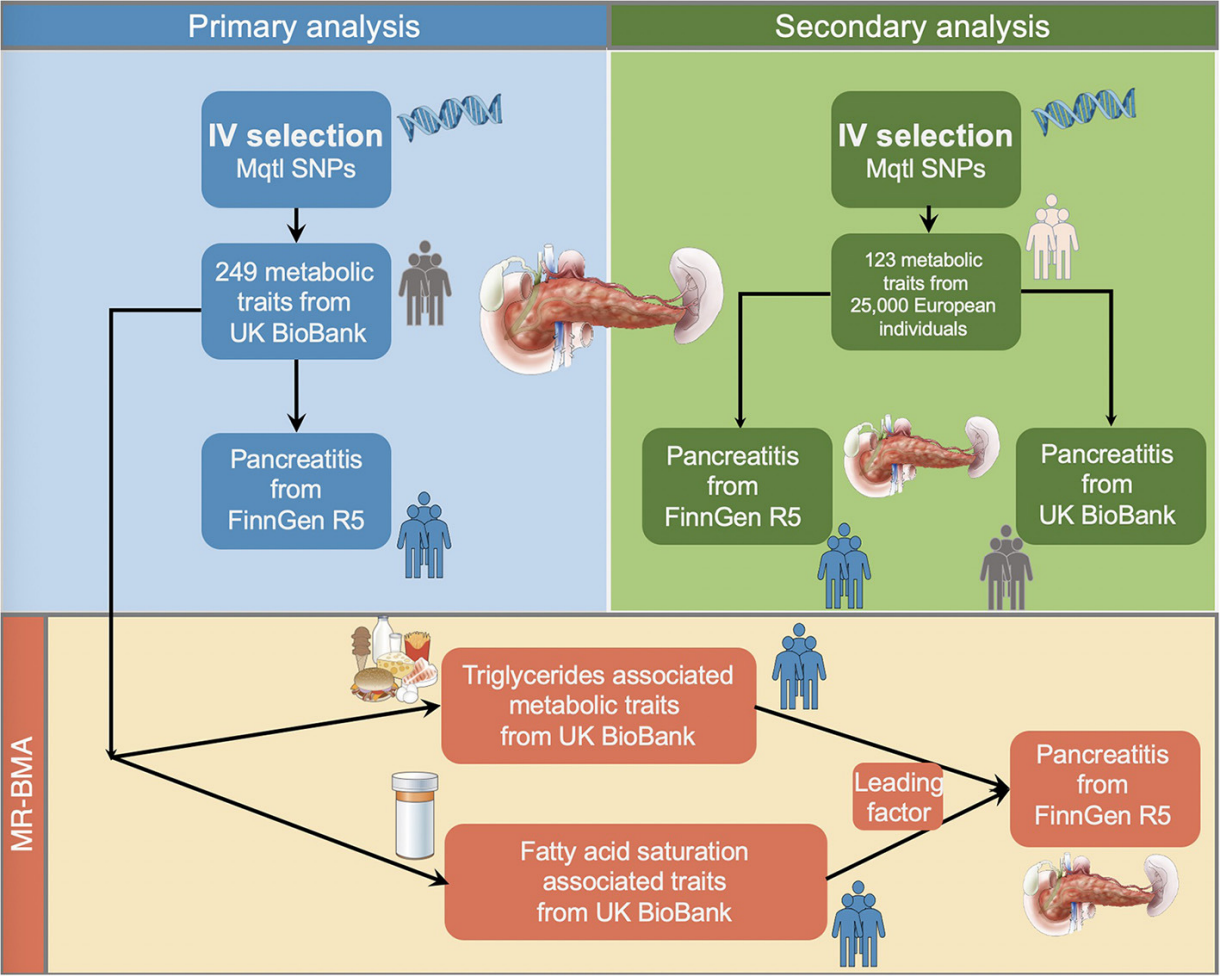
Schematic illustration of the study design. We performed the primary and secondary analysis using two distinct metabolites quantitative trait datasets on different pancreatitis datasets in the two-sample MR fashion. Next, we selected the metabolites with large numbers of shared single nucleotide polymorphisms for the Bayesian model averaging analyses to prioritize the predominating risk factor leading to susceptibility of pancreatitis. IVs, instrumental variables; Mqtl, metabolites quantitative trait loci.

This MR study used two plasma metabolite datasets to investigate their causal associations on the risk of pancreatitis. The outline of the study design is demonstrated in Figure 1. For the primary analysis, we selected the summary-level statistics for the exposures measured in UK Biobank population (met-d) and four pancreatitis datasets (acute pancreatitis, alcohol-induced acute pancreatitis, chronic pancreatitis, alcohol-induced chronic pancreatitis) from FinnGen Consortium Round 5 for two-sample MR. The analytical results were grouped into nine major subcategories [amino acids, low molecular weight metabolites, phospholipids, triglycerides, total lipids, (un)saturated fatty acids, cholesteryl esters, free cholesterol and apolipoprotein/lipoprotein]. We did a replication study by leveraging met-c data to for secondary validation using the six pancreatitis datasets (four FinnGen datasets together with acute pancreatitis and chronic pancreatitis from UK BioBank). In the above univariable MR, IVW, Weighted median and MR-Egger estimations were calculated for each analysis and we used the IVW method as the major readout to indicate causal associations. Finally, since the metabolic measurements demonstrated notable correlation (as they shared large numbers of genetic variants), we performed Bayesian model averaging MR (MR–BMA) in the met-d subcategory with large numbers of traits showing statistical significance, for the discovery of dominant metabolic factors driving the true causal effects over the others.

### Metabolite profiles (met-d) for primary analysis

Genetic datasets for 249 human metabolites (met-d) were generated from Nightingale Health Metabolic Biomarkers Phase 1 release GWAS studies involving 115,078 random-selected participants in UK Biobank (details in Supplementary methods). The biomarkers included measures of triglycerides and cholesterol metabolism, various fatty acid compositions and low molecular weight metabolites (such as amino acid, ketones and glycolysis metabolites). Moreover, among 14 lipoprotein subclasses, the triglycerides, phospholipids, total cholesterol, cholesterol esters, free cholesterol, and total lipid concentration were measured separately for each lipoprotein subclass. The SNPs were imputed based on HapMap-2 reference panel and GWAS analysis was performed by linear regression model after adjusting for age, sex, batch. After quality control, over 12.3 million SNPs were reserved for further analysis. The summary level data can be retrieved on MRBase database under the accession ID met-d.

### Metabolite profiles (met-c) for secondary analysis

Metabolite data were obtained from summary statistics of GWAS involving 123 blood metabolites (including lipoprotein subclass-specific lipids, amino acids, fatty acids, inflammatory glycoproteins, and others) measured by targeted NMR metabolomics on nearly 25,000 individuals. There are over 12 million SNPs detected, and we identified SNPs that were independently associated with each metabolite with linkage disequilibrium (LD) *R*^2^ < 0.001 and *p*-value < 5 × 10^−8^. The summary level data can be retrieved on MRBase database under the accession ID met-c.

### The selection of instrumental variables and data source

The instrumental variable (IVs) selection in MR analysis is based on three major assumptions: (1) the IVs are supposed to have direct associations with exposure (metabolite traits); (2) IVs are supposed to be unrelated with outcome and independent of any known or unknown confounding factors; (3) the effects of IVs on the outcomes are solely mediated by the exposures of interest. The selected IVs satisfy the conventional genome-wide significance (*P* < 5 × 10^−8^) with LD threshold of *R*^2^ < 0.001 within ±10,000 kilobase (kb) distance.

We used four pancreatitis GWAS summary statistics from FinnGen Round 5 (https://r5.finngen.fi/), including acute pancreatitis (3,022 cases and 195,144 controls, ICD-10, K80-K87, https://risteys.finngen.fi/phenocode/PANCREATITIS), alcohol-induced acute pancreatitis (457 cases and 218,335 controls, ICD-10, K85, https://risteys.finngen.fi/phenocode/ALCOPANCACU), chronic pancreatitis (1,737 cases and 195,144 controls, K86.00, K86.01, K86.08, K86.1, https://risteys.finngen.fi/phenocode/K11_CHRONPANC) and alcohol-induced chronic pancreatitis (977 cases and 217,815 controls, ICD-10, K86.00, K86.01, K86.08, https://risteys.finngen.fi/phenocode/ALCOPANCCHRON) for primary analysis. In the secondary analysis, we added two more recently published datasets from UK Biobank: acute pancreatitis (1,748 cases and 454,600 controls, Phecode ID 577.1, ICD-10 K85) and chronic pancreatitis (322 cases and 456,026 controls, PheCode 577.2, ICD-10 K86). The summary statistics can be downloaded from the GWAS catalog with accession numbers GCST90044204 (https://www.ebi.ac.uk/gwas/studies/GCST90044204) and GCST90044205 (https://www.ebi.ac.uk/gwas/studies/GCST90044205).

In FinnGen R5 data, 16,962,023 SNPs were analyzed using SAIGE software based on mixed-model logistic regression (https://github.com/weizhouUMICH/SAIGE/tree/finngen_r5_jk) with adjustments for sex, age, 10 Principal components (PCs) and genotyping batch. For the UK BioBank data, a generalized linear mixed model (GLMM)-based method named (fastGWA-GLMM) was utilized with adjustments for covariates including age, age2, sex, age × sex, age2 × sex and the top 20 PCs (8).

### Analysis with two-sample MR and Bayesian model averaging MR

The inverse-variance weighted (IVW) was used as the main method for causal inferences. The heterogeneity of the analyses was assessed with Cochran's Q values, I2 statistics, and the H statistics (9). We used MR-Egger and weighted median methods for sensitivity analyses. IVs with F-statistic ≥ 10 were considered as effective IVs (10).

MR-BMA was used in the subcategory showing large numbers of metabolic traits with statistical significance. Compared with conventional multivariable MR, the MR-BMA method is particularly useful to investigate high-dimensional datasets (i.e., metabolites) with remarkable genetic correlation (11, 12). The rationale is that subgroups of metabolites sharing large numbers of genetic variants may act together on the same causal pathway. With MR-BMA, the related metabolites can be disentangled to identify the predominate traits with causal signals.

After univariable MR, we identified the subcategory (fatty acid saturation and triglycerides) showing large numbers of metabolic traits with statistical significance. The traits with a *p*-value < 0.05 in IVW method were included for MR-BMA. After strict clumping of combined SNPs (*R*^2^ < 0.001 in 10,000 kb distance), we calculated the posterior probability (PP) for each specific model. We used marginal inclusion probability (MIP), which stands for the sum of the PP over all possible models, to rank the traits. Those with the highest MIP rank are suggestive of the strongest “true causal” candidates. Furthermore, the model-averaged causal estimate (MACE), which reflects the average direct effect of each metabolic traits on the outcomes, was computed. Finally, the best models by the PP values (with a PP threshold of 0.02) of the individual models were prioritized. Invalid instruments detected as outliers according to the Q statistics and Cook's distance were removed and we repeated the aforementioned calculation step to get the best model and estimation values (PP, MIP, causal estimates and MACE).

### Statistical analysis and tools

The MR results are 2-sided, and a *p*-value < 0.05 was considered as statistically significant for primary and secondary analyses. We avoid using multiple testing *p*-value corrections as many of the traits were not independent as they shared large numbers of genetic variants, and thereby multiple testing might filter out valid exposures. To overcome this issue, we performed the MR-BMA analysis using all related traits with a *p*-value < 0.05 as the input, which subsequently enble us to identify the independent dominant traits. All the analyses were performed on R platform (version 4.0.2). The “TwoSampleMR” (0.5.5) and “Mendelian Randomization” (0.5.0) packages were used for statistical analyses in two-sample MR and “ggplot2” package was used for data visualizations (13–15). The MR-BMA were conducted with R-code deposited in github (https://github.com/verena-zuber/demo_AMD).

## Results

### Primary analysis results using two-sample MR approach

**Figure 2:**
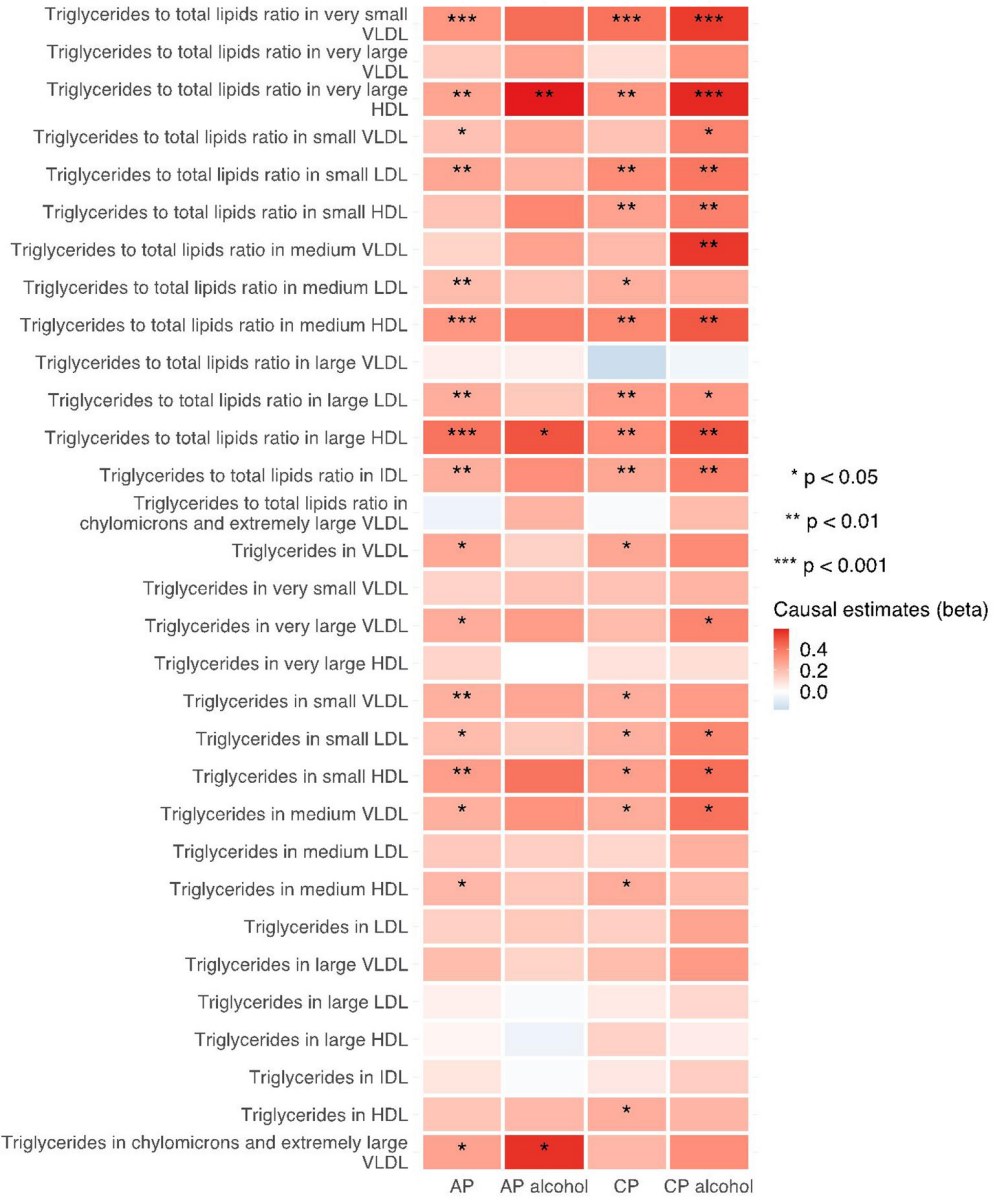
Heatmap showing the IVW causal estimates of triglycerides related traits in met-d on pancreatitis using two-sample MR. AP, acute pancreatitis; CP, chronic pancreatitis; IVW, inverse-variance weighted; VLDL, very-low-density lipoprotein; LDL, low-density lipoprotein; IDL, intermediate-density lipoprotein; HDL, high-density lipoprotein.

**Figure 3:**
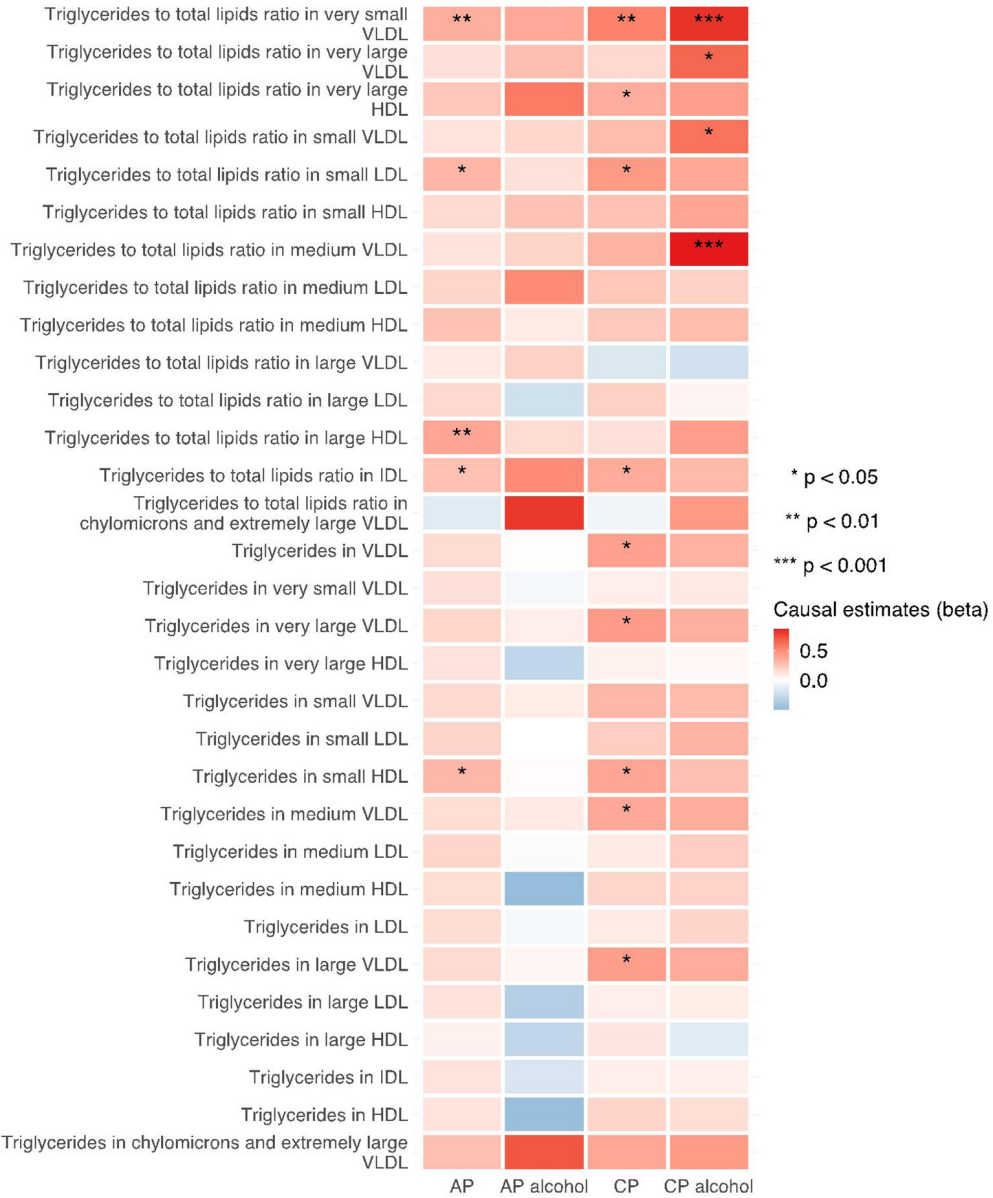
Heatmap showing the weighted median causal estimates of triglycerides related traits in met-d on pancreatitis using two-sample MR. AP, acute pancreatitis; CP, chronic pancreatitis; IVW, inverse-variance weighted; VLDL, very-low-density lipoprotein; LDL, low-density lipoprotein; IDL, intermediate-density lipoprotein; HDL, high-density lipoprotein.

**Figure 4:**
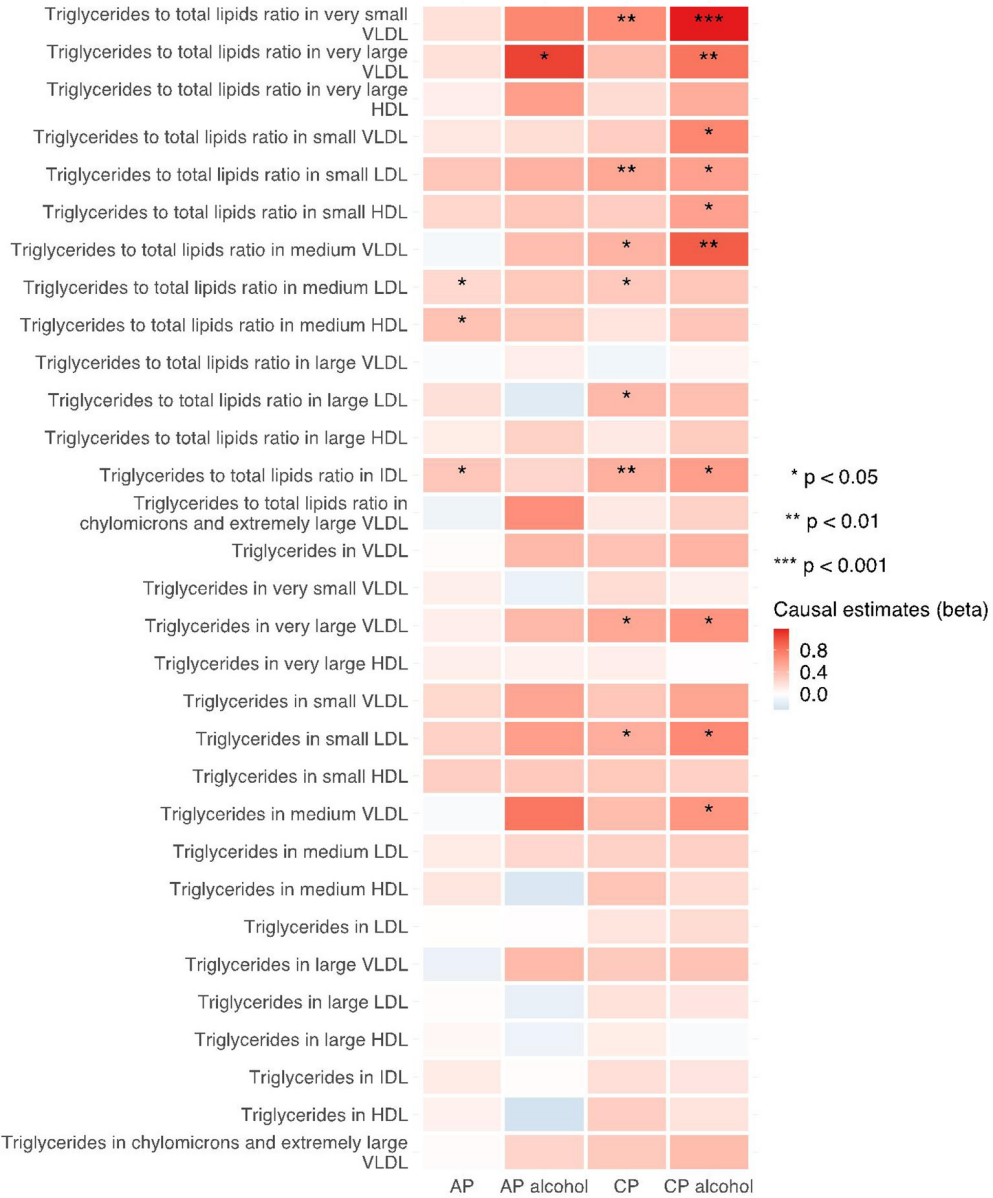
Heatmap showing the MR-egger causal estimates of triglycerides related traits in met-d on pancreatitis using two-sample MR. AP, acute pancreatitis; CP, chronic pancreatitis; IVW, inverse-variance weighted; VLDL, very-low-density lipoprotein; LDL, low-density lipoprotein; IDL, intermediate-density lipoprotein; HDL, high-density lipoprotein.

The 249 metabolites for univariable MR in primary analysis can be subdivided into nine major categories, including amino acids, low molecular weight metabolites, phospholipids, triglycerides, total lipids, (un)saturated fatty acids, cholesteryl esters, free cholesterol and apolipoprotein/lipoprotein. We observed universal positive correlation trends between triglycerides-related traits and the risk of all pancreatitis subtypes. Among them, triglycerides to total lipids ratio in very small VLDL and large HDL demonstrated significant associations with all four types of pancreatitis. Although most of the traits became statistically insignificant in weighted median and MR-Egger results, the positive correlation trends remained. Moreover, triglycerides to total lipids ratio in very small VLDL and small HDL showed a strong positive causal association with the increased risk of alcohol-induced chronic pancreatitis among all three MR methods (shown in Figures 2–4).

**Figure 5:**
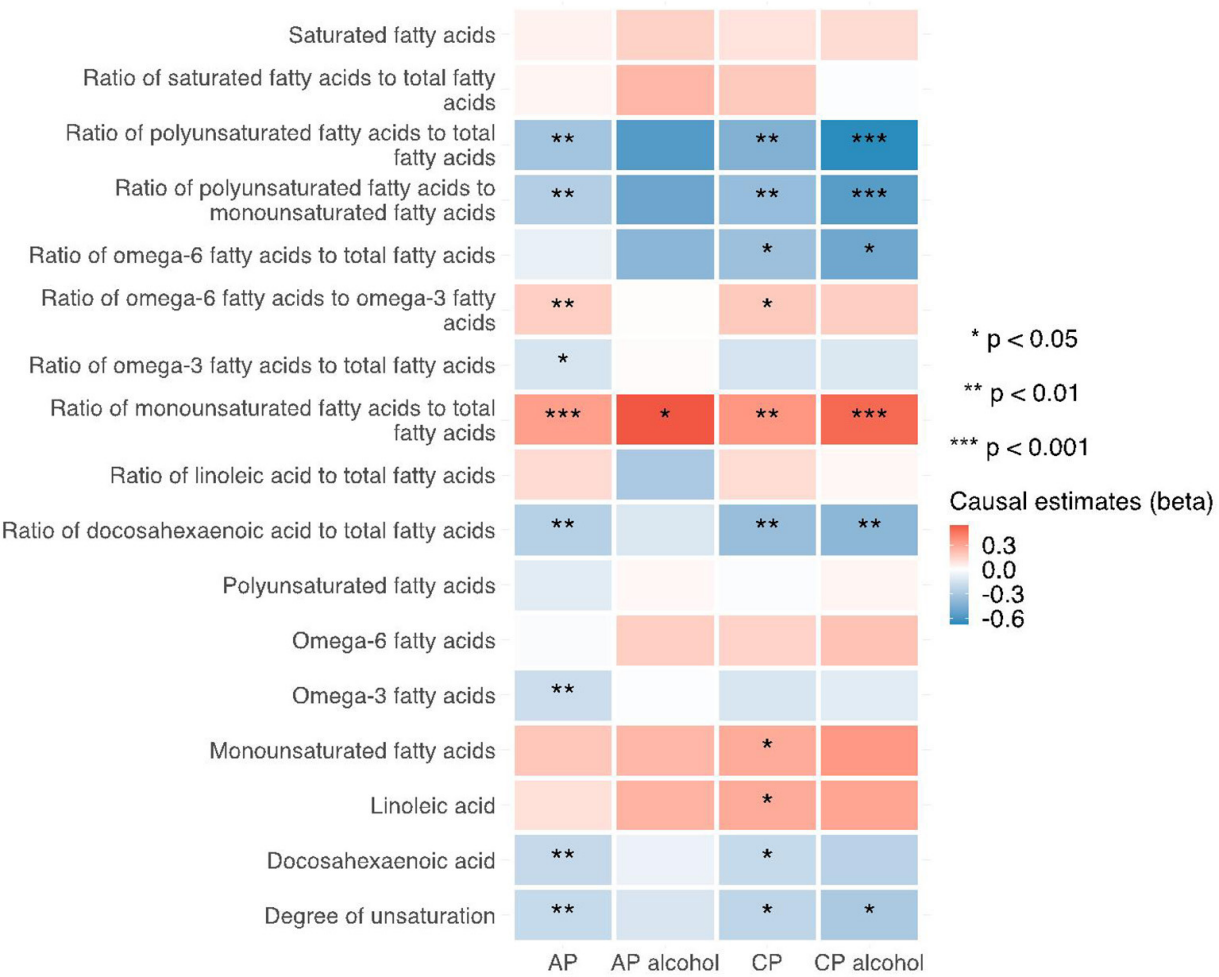
Heatmap showing the IVW causal estimates of traits related with fatty acid (un)saturation in met-d on pancreatitis using two-sample MR. AP, acute pancreatitis; CP, chronic pancreatitis; IVW, inverse-variance weighted.

**Figure 6:**
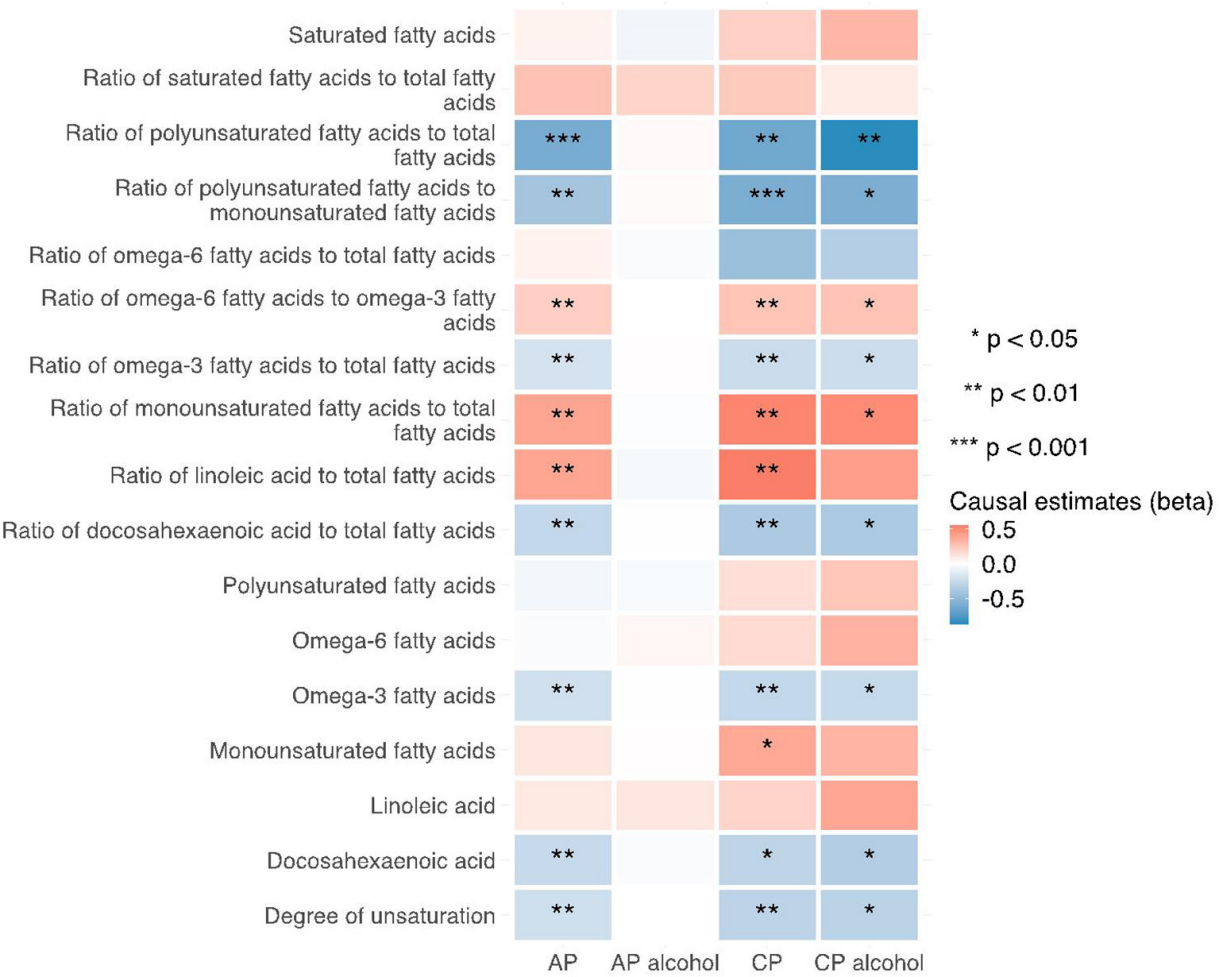
Heatmap showing the weighted median causal estimates of traits related with fatty acid (un)saturation in met-d on pancreatitis using two-sample MR. AP, acute pancreatitis; CP, chronic pancreatitis; IVW, inverse-variance weighted.

**Figure 7:**
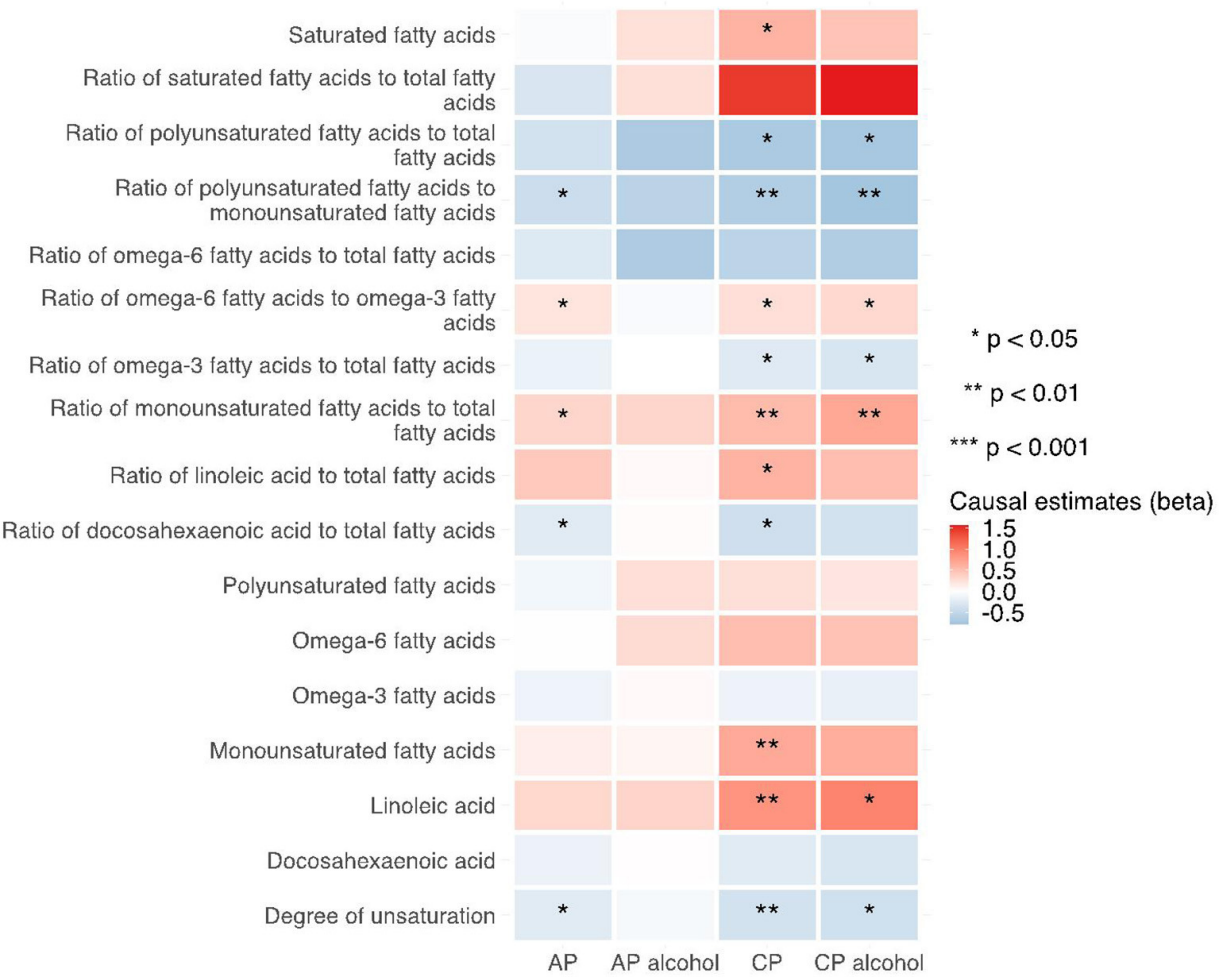
Heatmap showing the MR-egger causal estimates of traits related with fatty acid (un)saturation in met-d on pancreatitis using two-sample MR. AP, acute pancreatitis; CP, chronic pancreatitis; IVW, inverse-variance weighted.

Notably, various traits related to saturation levels of fatty acids were either positive or inversely correlated with the risk of pancreatitis. Among them, the ratio of polyunsaturated fatty acid to total fatty acids, the ratio of polyunsaturated fatty acids to monounsaturated fatty acids, docosahexaenoic acid (DHA, an omega-3 fatty acid), the ratio of docosahexaenoic acid to total fatty acids, and the degree of unsaturation are inversely associated with acute pancreatitis, chronic pancreatitis, and alcohol-induced chronic pancreatitis; whereas the ratio of omega-6 fatty acids to omega-3 fatty acids and the ratio of monounsaturated fatty acids to total fatty acids showed causal effects on the increased risk of several pancreatitis. The sensitivity analysis results with weight median and MR-Egger methods identified a similar association trend as the IVW method (shown in Figures 5–7). The details for effect values in associations, causal effects for each IV, heterogeneity and pleiotropic testing results were shown in Supplementary Tables 1–4.

The heatmaps of other metabolic traits were displayed in Supplementary Figures 1–7.

### Secondary analysis results using two-sample MR approach

We replicated the MR analyses with met-c datasets and found that traits related to triglycerides (such as triglycerides in medium VLDL, triglycerides in very large VLDL and triglycerides in chylomicrons and largest VLDL particles) are associated with the increased risk of pancreatitis. Interestingly, traits indicating a high degree of unsaturation (including the ratio of bisallylic groups to double bonds, the ratio of bisallylic groups to total fatty acids, the average number of double bonds in a fatty acid chain and omega-6 fatty acids) showed protective effects on the reduced risk of pancreatitis. The details for effect values in associations, causal effects for each IV, heterogeneity and pleiotropic testing results were shown in Supplementary Tables 1–4.

### MR-BMA analysis to identify leading traits on various types of pancreatitis

We applied MR-BMA method in triglycerides and (un)saturated fatty acid categories with SNPs used in primary analyses. We used acute pancreatitis and chronic pancreatitis as the major outcomes for MR-BMA, as these two datasets include larger numbers of cases.

For the triglycerides category, we identified 130 SNPs and 129 IVs for acute pancreatitis and chronic pancreatitis, respectively. After correcting for outliers, 1 SNP (*CETP* gene) was removed in acute pancreatitis. We observed that triglycerides in HDLs in different sizes are the leading factors of acute pancreatitis risk. For chronic pancreatitis, all three traits related with acute pancreatitis as well as triglycerides in small HDL showed causal effects (Table 1).

**Table 1:** Ranking of triglycerides related traits for acute pancreatitis and chronic pancreatitis using MR-BMA.

**Risk factor or model**	**Ranking by MIP**	**MIP**	**MACE**	**Ranking by PP**	**PP**	**Causal estimates**	***p*-value**
**Acute pancreatitis**							
**Model averaging using 130 SNPs**							
Triglycerides to total lipids ratio in very small VLDL	1	0.226	0.075	1	0.143	0.293	0.020
Triglycerides to total lipids ratio in very large HDL	2	0.137	0.041	3	0.075	0.334	0.040
**Model averaging using 129 SNPs (exclude CETP)**							
Triglycerides to total lipids ratio in large HDL	1	0.425	0.201	1	0.192	0.371	0.010
Triglycerides to total lipids ratio in very large HDL	2	0.206	0.026	2	0.075	0.337	0.030
Triglycerides to total lipids ratio in medium HDL	3	0.116	0.09	3	0.054	0.316	0.040
**Chronic pancreatitis**							
**Model averaging using 129 SNPs**							
Triglycerides to total lipids ratio in medium HDL	1	0.196	0.065	2	0.108	0.361	0.030
Triglycerides to total lipids ratio in large HDL	2	0.195	0.065	1	0.109	0.365	0.020
Triglycerides to total lipids ratio in very large HDL	3	0.169	0.057	3	0.088	0.373	0.020
Triglycerides in small HDL	4	0.155	0.051	4	0.081	0.362	0.010

MIP, marginal inclusion probability; MR, mendelian randomization; MR-BMA, MR based on Bayesian model averaging; PP, posterior probability; HDL, high-density lipoprotein; TG, triglycerides; SNP, Single Nucleotide Polymorphism.

For (un)saturated fatty acid category, 83 SNPs and 96 SNPs were used as IVs for acute and chronic pancreatitis after clumping. One SNP each (one SNP in *FADS2* gene and one SNP for *MYRF* gene) was detected as the outlier and removed in the following analysis. The degree of unsaturation is the only dominant trait showing inverse association; while the level of monosaturated fatty acid and the ratio of polyunsaturated fatty acids to monosaturated fatty acids were the dominant traits, showing deleterious and protective impacts on the risk of chronic pancreatitis (Table 2).

**Table 2:** Ranking of traits indicative of the level of fatty acid saturation for acute pancreatitis and chronic pancreatitis using MR-BMA.

**Risk factor or model**	**Ranking by MIP**	**MIP**	**MACE**	**Ranking by PP**	**PP**	**Causal estimates**	***p*-value**
**Acute pancreatitis**							
**Model averaging using 83 SNPs**							
Degree of unsaturation	1	0.274	−0.064	1	0.209	−0.232	0.010
Ratio of docosahexaenoic acid to total fatty acids	3	0.187	−0.041	2	0.135	−0.265	0.020
**Model averaging using 82 SNPs (exclude FADS2)**							
Degree of unsaturation	1	0.218	−0.051	1	0.162	−0.237	0.040
**Chronic pancreatitis**							
**Model averaging using 96 SNPs**							
Degree of unsaturation	1	0.247	−0.072	1	0.151	−0.285	0.010
Ratio of monounsaturated fatty acids to total fatty acids	2	0.224	0.065	2	0.143	0.315	0.040
Ratio of polyunsaturated fatty acids to monounsaturated fatty acids	3	0.195	−0.056	3	0.122	−0.326	0.040
Ratio of docosahexaenoic acid to total fatty acids	4	0.182	−0.053	4	0.108	−0.328	0.050
**Model averaging using 95 SNPs (exclude MYRF)**							
Ratio of polyunsaturated fatty acids to monounsaturated fatty acids	3	0.161	−0.032	4	0.101	−0.22	0.050
Ratio of monounsaturated fatty acids to total fatty acids	5	0.154	0.029	5	0.097	0.212	0.030

MIP, marginal inclusion probability; MR, mendelian randomization; MR-BMA, MR based on Bayesian model averaging; PP, posterior probability; SNP, Single Nucleotide Polymorphism.

## Discussion

This study gave concrete evidence showing the strong causal effects of triglycerides, particularly triglycerides in HDL particles, on the risk of pancreatitis. Moreover, we observed traits related with increased fatty acid unsaturation levels were negatively associated with pancreatitis risk. MR-BMA results indicated that the degree of unsaturation as well as the level of monounsaturated fatty acid and the ratio of polyunsaturated fatty acid to monounsaturated fatty acid level were the leading factors in acute and chronic pancreatitis, respectively.

### Triglycerides and pancreatitis

High triglycerides levels have been commonly accepted as the major risk factor. For example, two prospective cohorts involving over 118,000 individuals in Copenhagen city suggested that the elevated level of triglycerides was the mediator of high BMI in inducing pancreatitis (16). A recent meta-analysis also showed that elevated level of triglycerides was strongly correlated with a worse prognosis of acute pancreatitis, such as higher mortality rate and higher risk of developing systemic inflammatory response syndrome (17). Also, patients with chronic pancreatitis had higher levels of triglycerides (18). One MR study also revealed that genetically predicted high triglycerides level was associated with increased risk of chronic pancreatitis, with an ORs equal to 1.47 (7).

Here, we provided consistent results showing that multiple triglycerides traits (especially triglycerides to total lipid ratio in very small VLDL, very large HDL, medium HDL, large HDL, and small HDL) were associated with increased risk of pancreatitis. After correction for potential pleiotropic effects, MR-BMA results indicated that triglycerides to total lipid ratio in different types of HDL particles were considered as the predominant causal factors in the risk of both acute and chronic pancreatitis. These results are in agreement with preceding findings, indicating the levels of triglycerides (TG) to high-density lipoprotein (HDL) cholesterol ratio is a potential biomarker in predicting the severity of acute pancreatitis (19).

### Saturated/unsaturated fatty acids and pancreatitis

There are several lines of evidence showing that the supplementation of unsaturated fatty acid, especially omega-3 fatty acids, might play beneficial roles in the development and prognosis of acute pancreatitis. One animal study done in experimental acute pancreatitis rat model showed that rats intravenously treated with fish oil (mainly omega-3 fatty acids) had significant higher level of interleukin (IL)-10 (an anti-inflammatory cytokine) together with fewer episodes of respiratory dysfunction (20). The lactate dehydrogenase levels as well as pro-inflammatory cytokines (such as IL-6 and tumor necrosis factor-alpha [TNF-alpha]) were decreased with the administration of omega-3 fatty acids (21). This might be due to that the supplementation with omega-3 fatty acids can attenuate pancreatic and pulmonary macrophage mediated inflammatory response and the downregulation of Toll-like receptor 4 (TLR4)/nuclear factor κB p56 (NF-κBp56) signaling (22, 23). In all, these results together provided experimental evidence suggesting potential beneficial role of polyunsaturated fatty acid, especially omega-3 fatty acids, in the inhibition of exacerbation of inflammatory response in acute pancreatitis.

Observational and prospective cohorts also provided evidence demonstrating beneficial role of omega-3 fatty acids in the treatment of pancreatitis. Two meta-analyses involving RCTs only with omega-3 fatty acid administration demonstrated significant reduced risk of mortality, infectious complications, length of hospital stay and new-organ failure without safety issue reported (24, 25). A very recent RCT study also showed that intravenous omega-3 fatty acids administration also achieved better clinical outcomes in terms of lowering total blood leukocyte number, CRP, IL-8, multiple organ dysfunction score, sequential organ failure assessment score, and systemic inflammatory response syndrome (26). A case study in pregnant women with familial hypertriglyceridemia and type 2 diabetes mellitus (high-risk group of developing acute pancreatitis) suggested dietary supplementation of omega-3 fatty acids was of value in preventing pancreatitis (27). Here, we found that traits related with elevated levels of unsaturation (the ratio of polyunsaturated fatty acids to total fatty acids, the ratio of polyunsaturated fatty acids to monounsaturated fatty acids, the ratio of DHA to total fatty acids, DHA level, the degree of unsaturation) showed protective effects; while traits related with elevated levels of saturation (the ratio of omega-6 fatty acids to omega-3 fatty acids, the ratio of monounsaturated fatty acids to total fatty acids) showed negative effects. Replication analysis also described that the degree of unsaturation indicated by the ratio of bisallylic groups to total fatty acids, the ratio of bisallylic groups to double bonds, showed inverse associations. MR-BMA results further demonstrated that the degree of unsaturation was the only dominant factor in showing inverse association with acute pancreatitis. In chronic pancreatitis, the ratio of polyunsaturated fatty acid to monounsaturated fatty acid stood out as protective factor, while the ratio of monounsaturated fatty acid suggested a negative impact. These results indicated that dietary supplementation to increase polyunsaturated fatty acid levels might be a potential cost-effective therapeutic strategy in the primary prevention of pancreatitis.

### Limitations

First, all the exposure and outcome data are based on European populations. We need to be cautious when making inferences in other populations. Second, the MR method assumed a lifetime exposure of high circulating metabolites level in the blood, which is hardly to be true in reality. Therefore, the direction of causal effect might be more important and the calculated estimates should not be overestimated. Third, due to the MR study design, we are unable to discover a possible U-shape correlation between plasma metabolic traits on pancreatitis risk. Lastly, though gender and age have been considered as co-variates for individual GWAS, these factors as well as other co-variates are well worth taken into consideration when the individual data is available.

## Conclusion

Our comprehensive MR study reveals that elevated triglycerides levels and reduced degree of unsaturation in fatty acids were causally associated with the increased risk of various pancreatitis. Dietary supplementation of polyunsaturated fatty acids (especially omega-3 fatty acids) might be a useful therapeutic strategy in the prevention of pancreatitis.

## Data availability statement

The datasets presented in this study can be found in online repositories. The names of the repository/repositories and accession number(s) can be found in the article/Supplementary material.

## Ethics statement

Ethical review and approval were waived for this study, due to the data from the public database. The ethical permits have been obtained by individual studies from the Local Ethical Committee included in the analyses. Informed consent was obtained from all subjects involved in the study in the public database.

## Author contributions

JM, ZL, LJ, XB, and YF: conception and design. XB and YF: administrative support. JM, ZL, and XB: provision of study materials or patients. JM, ZL, ML, XW, and XB: collection and assembly of data. JM, ZL, LJ, NZ, ZW, and XB: data analysis and interpretation. All authors: manuscript writing and final approval of manuscript.

## Funding

This study was supported by the CAMS Innovation Fund for Medical Sciences (2021-I2M-1-013), National High Level Hospital Clinical Research Funding and Fundamental Research Funds (2022-PUMCH-A-074 and 2022-PUMCH-A-177), National Key Clinical Specialist Construction Project (ZK108000) and National Natural Science Foundation of China, Joint Fund Project (Integrated Project Grant No. U20A6001).

## Conflict of interest

The authors declare that the research was conducted in the absence of any commercial or financial relationships that could be construed as a potential conflict of interest.

## Publisher's note

All claims expressed in this article are solely those of the authors and do not necessarily represent those of their affiliated organizations, or those of the publisher, the editors and the reviewers. Any product that may be evaluated in this article, or claim that may be made by its manufacturer, is not guaranteed or endorsed by the publisher.
