# Supplementary figures and images for: Mendelian randomization in blood metabolites identifies triglycerides and fatty acids saturation level as associated traits linked to pancreatitis risk
Source: Front Nutr. 2022 Oct 10;9:1021942. doi: 10.3389/fnut.2022.1021942 (PMC9589364; doi:10.3389/fnut.2022.1021942)

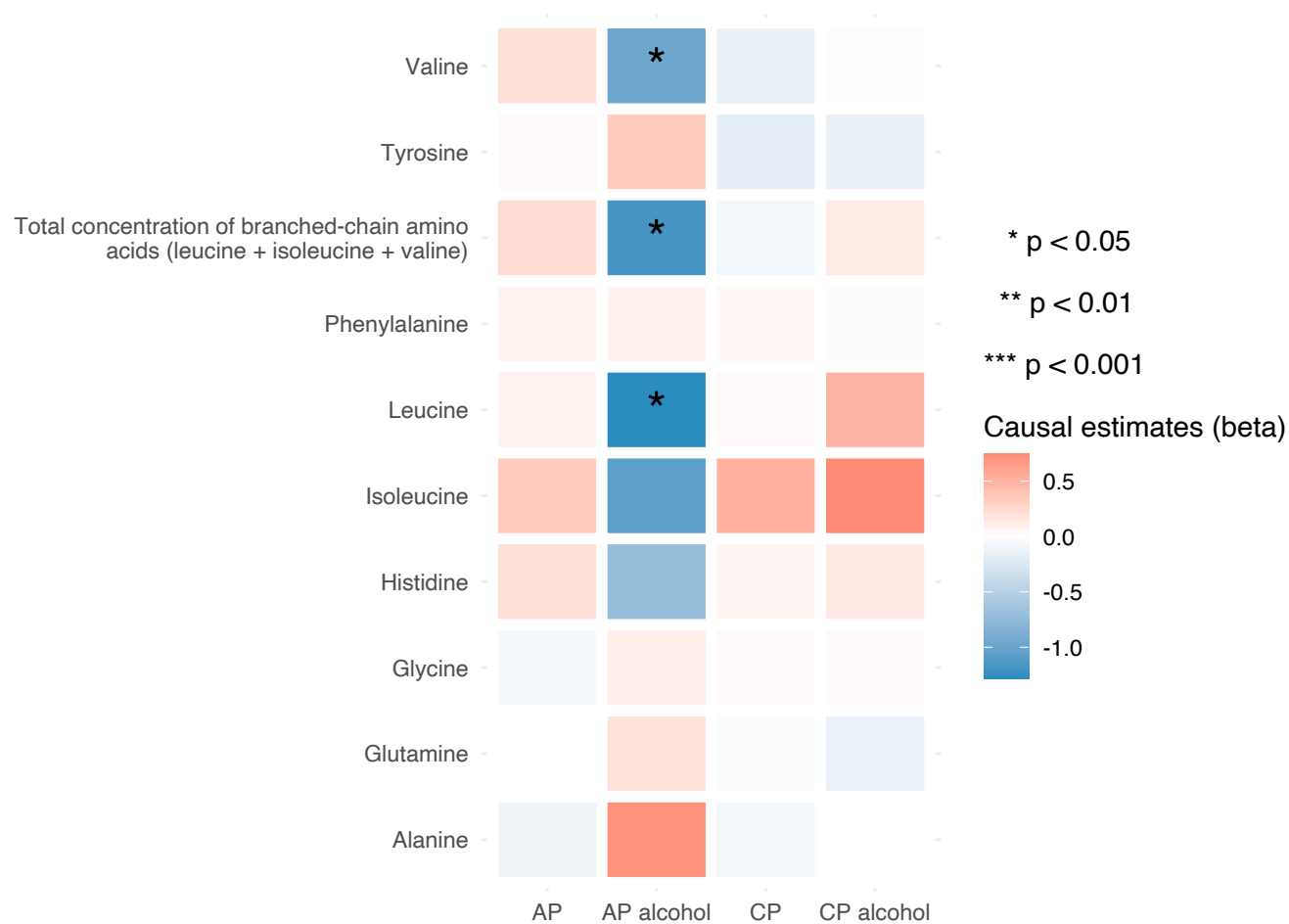

Supplement: Supplementary file 1 [file Data_Sheet_1.PDF]

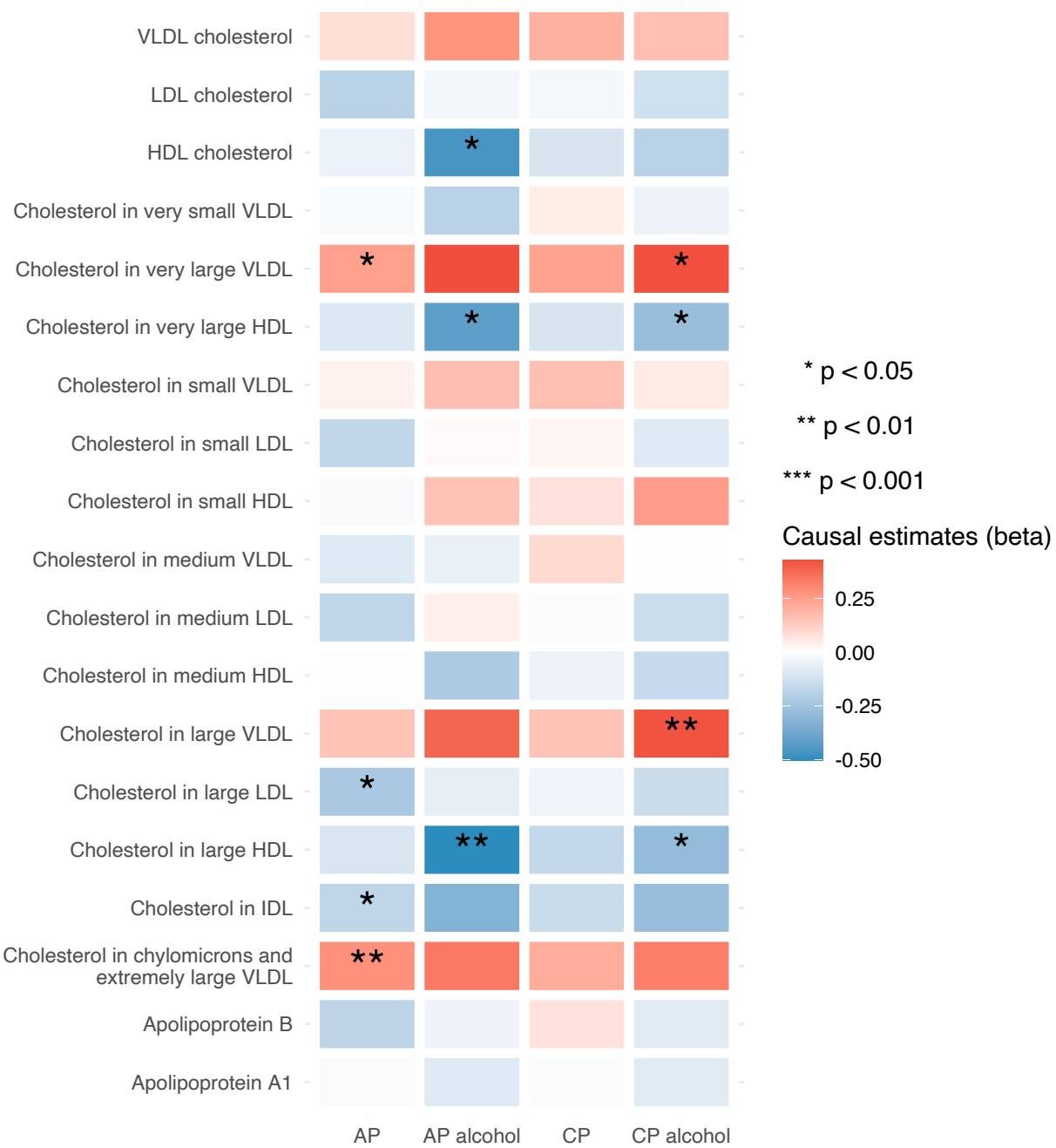

Supplement: Supplementary file 2 [file Data_Sheet_2.PDF]

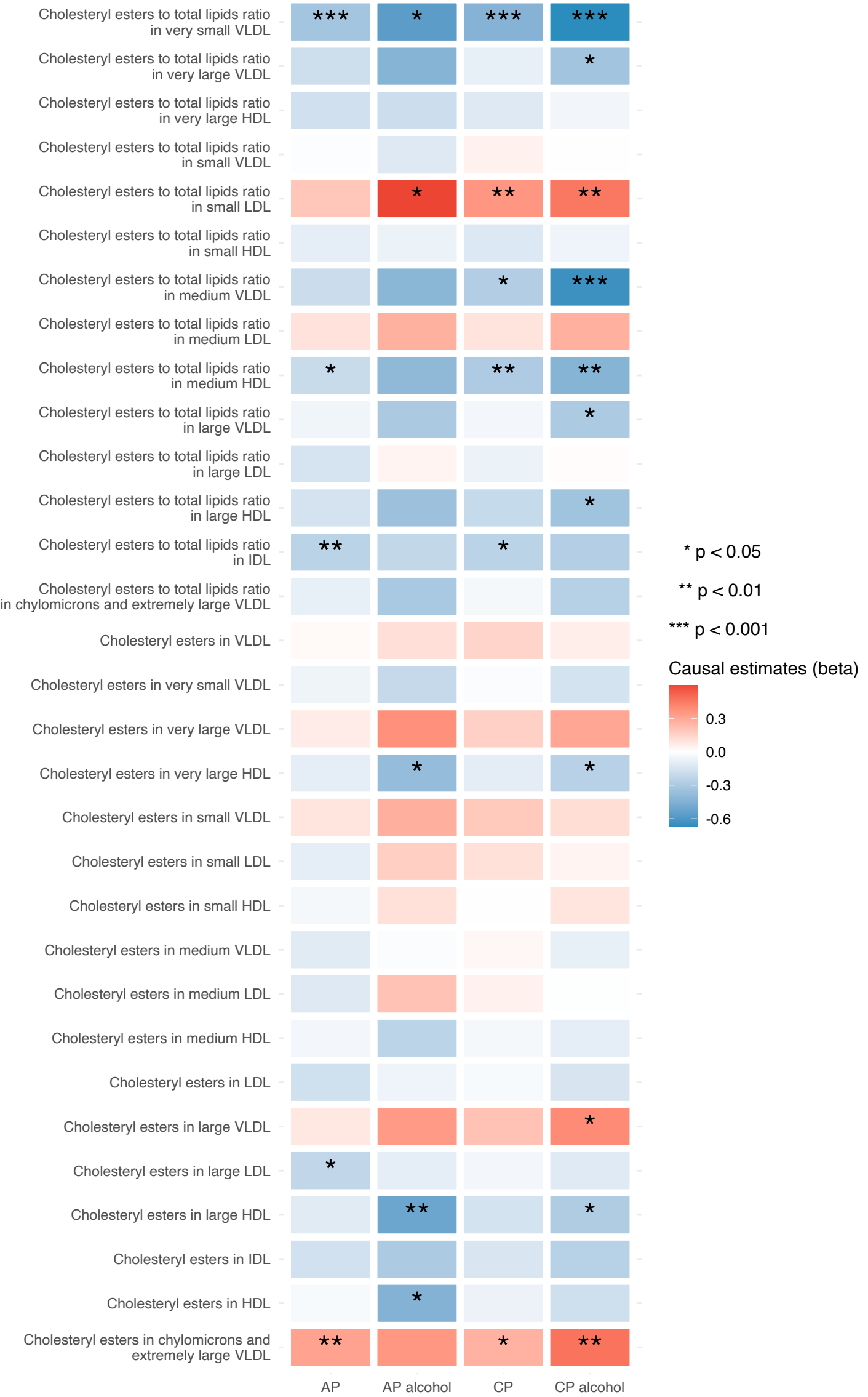

Supplement: Supplementary file 3 [file Data_Sheet_3.PDF]

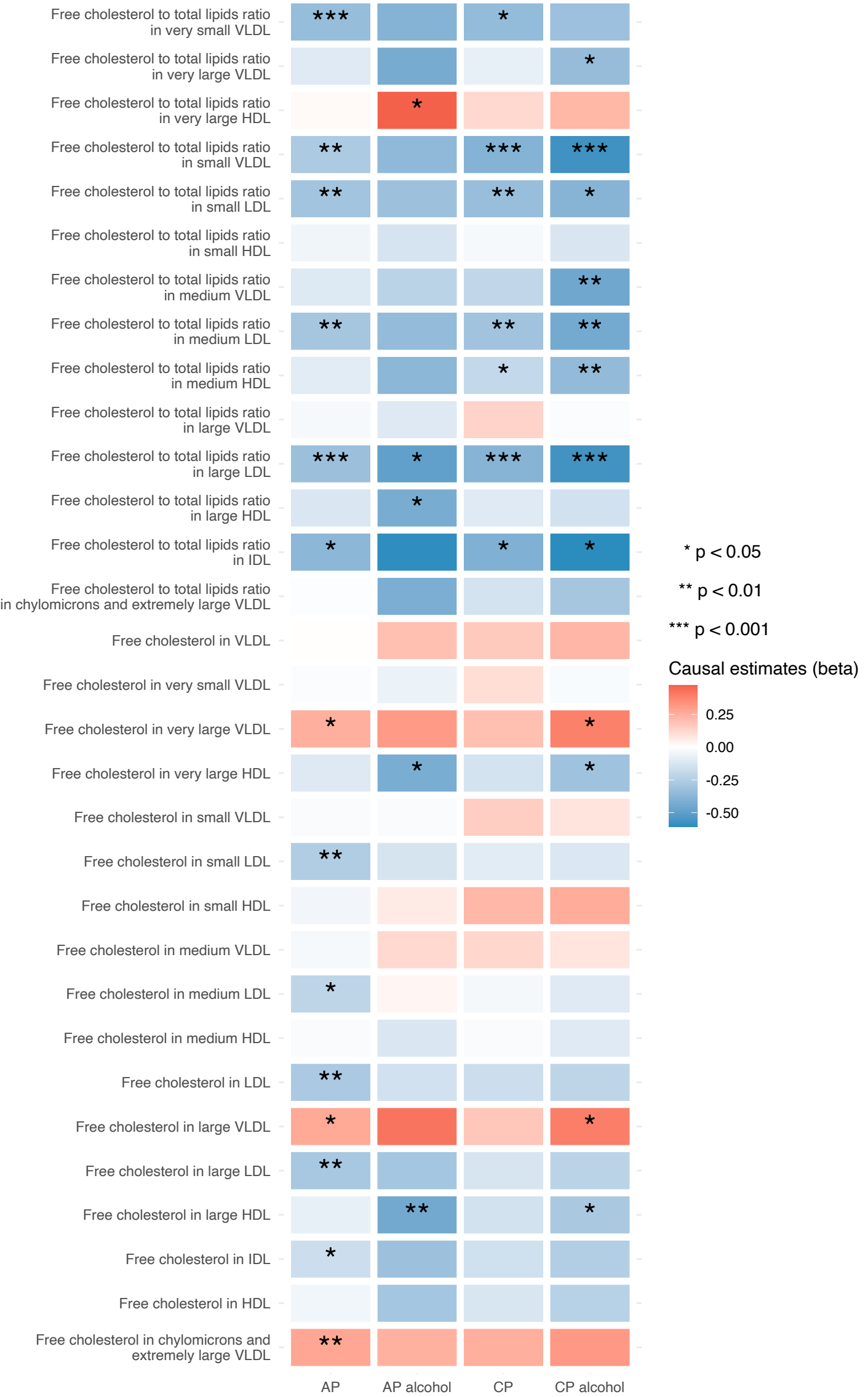

Supplement: Supplementary file 4 [file Data_Sheet_4.PDF]

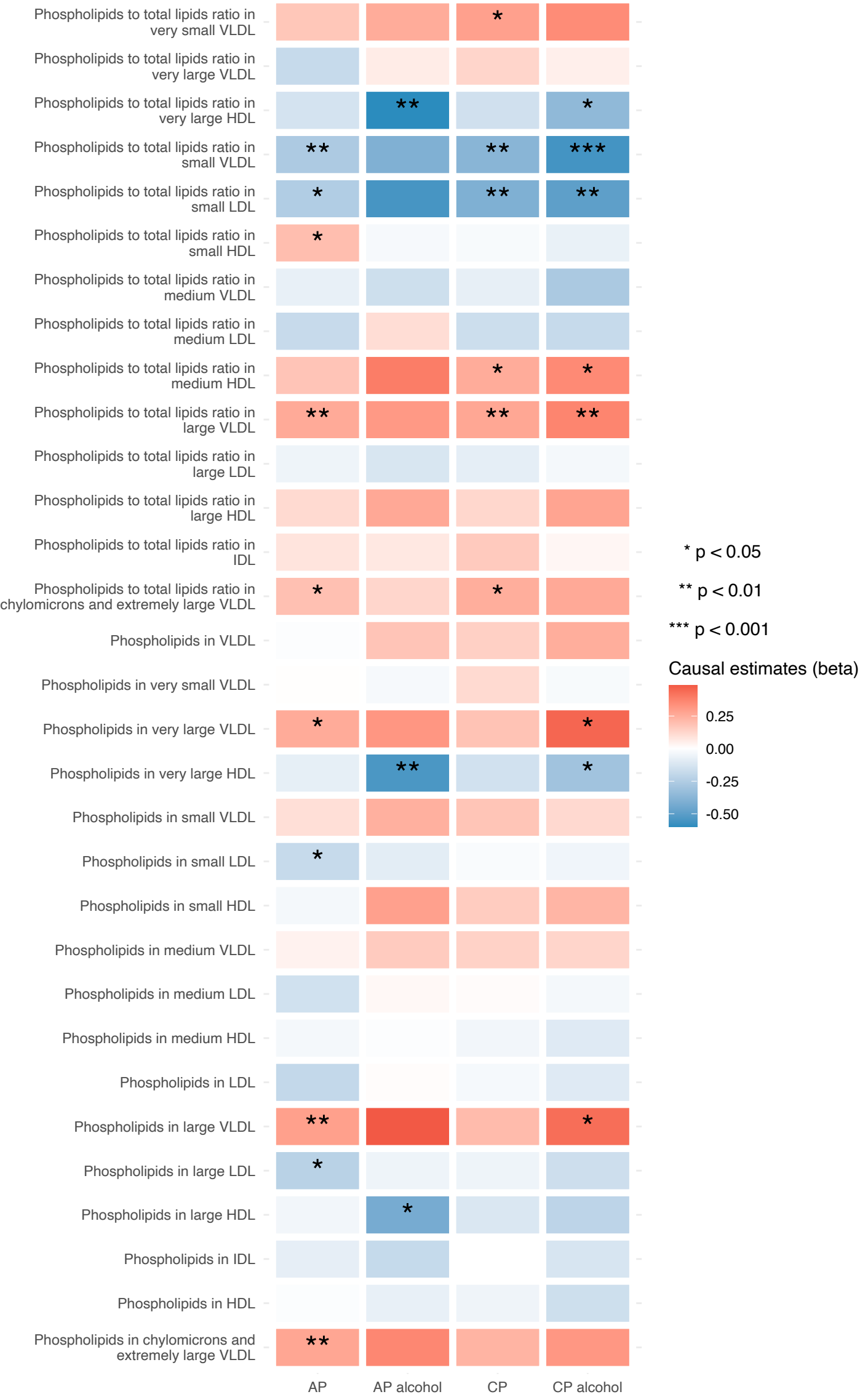

Supplement: Supplementary file 5 [file Data_Sheet_5.PDF]

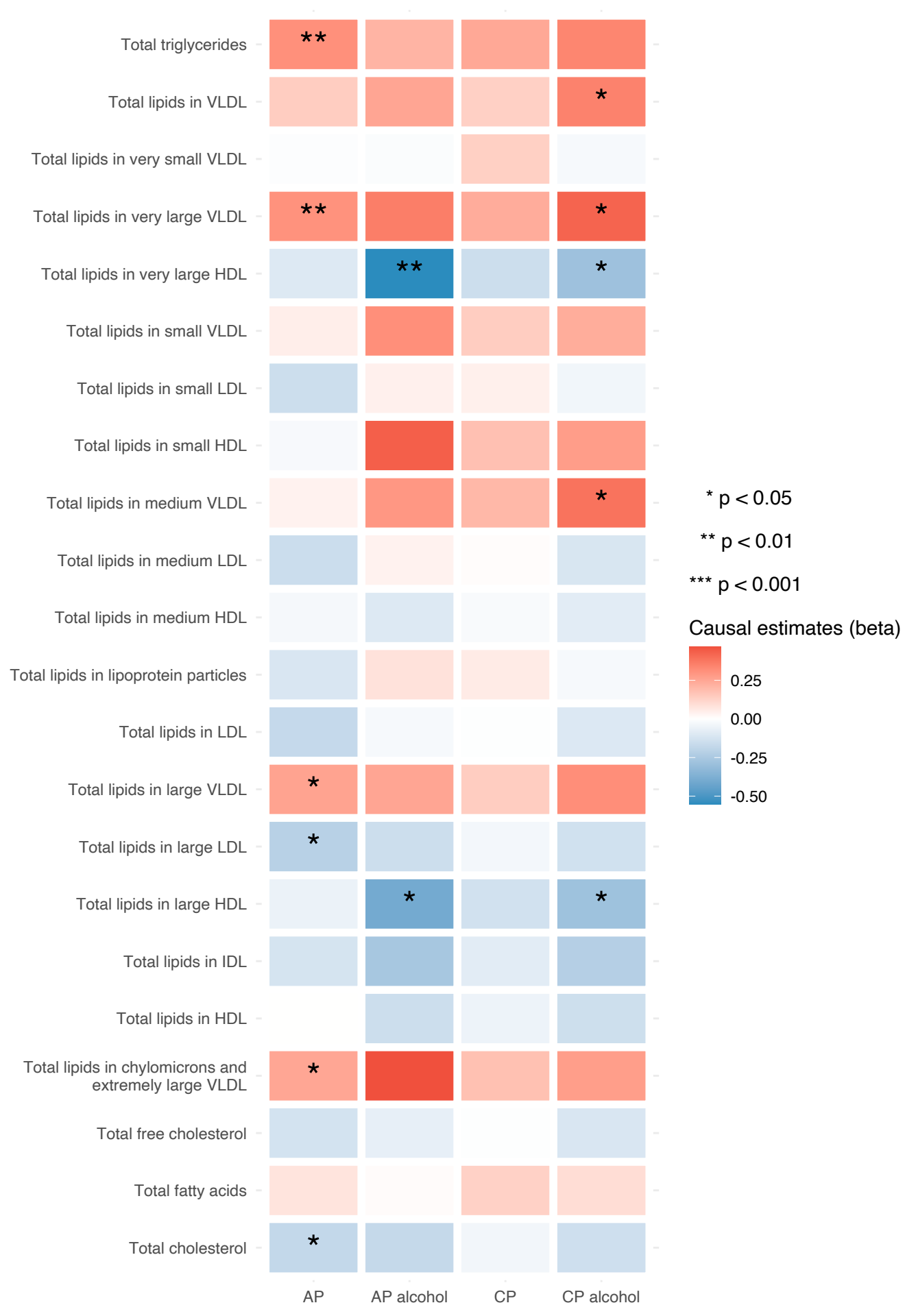

Supplement: Supplementary file 6 [file Data_Sheet_6.PDF]

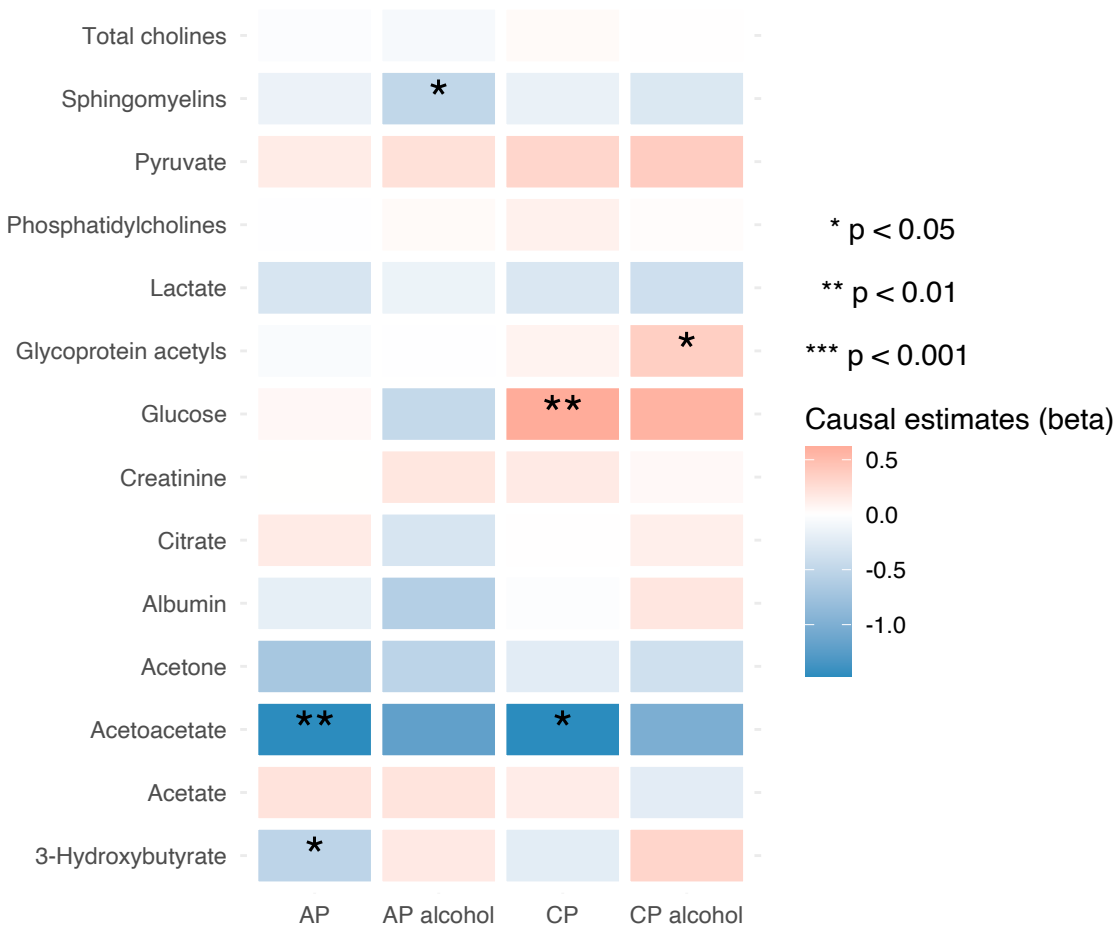

Supplement: Supplementary file 7 [file Data_Sheet_7.PDF]
