# Supplementary material for: Mendelian randomization in blood metabolites identifies triglycerides and fatty acids saturation level as associated traits linked to pancreatitis risk
Source: Front Nutr. 2022 Oct 10;9:1021942. doi: 10.3389/fnut.2022.1021942 (PMC9589364; doi:10.3389/fnut.2022.1021942)
Supplement: Supplementary file 16 [file Table_9.DOCX]

**Supplementary materials**

**Supplementary Methods**

**The metabolic traits measurements in Nightingale Health**

The non-fasting baseline plasma samples were collected in EDTA tubes and the metabolic profiling was measured based on high-throughput nuclear magnetic resonance (NMR), with 168 metabolites measured in absolute concentrations (unit, mmol/L) and 81 measures in ratios (https://biobank.ndph.ox.ac.uk/ukb/label.cgi?id=220). The samples were prepared directly in 96 well-plates by UK Biobank with over 85 µL plasma aliquoted in each well. Next, the plasma samples were shipped to Nightingale Health’s laboratories in the 96-well plates on dry ice in sample batches of ~5,000-20,000. The details of NMR profiling and experimental settings (developed by Nightingale Health Ltd.) have been described in previous studies (Pasi Soininen *et al*, 2015; Ari V Ahola-Olli *et al*, 2019). In brief, two NMR spectra were recorded using a 500 MHz NMR spectrometer (Bruker AVANCE IIIHD), with the first pre-saturated proton spectrum detecting proteins and lipids within various lipoprotein particles and the second T2-relaxation-filtered spectrum detecting low-molecular-weight metabolites. The metabolic biomarker quantifications were measured using Nightingale Health’s proprietary software (Nightingale Health biomarker quantification library 2020). BOLT-LMM (linear mixed model) was used to account for population structure in UK Biobank, with further adjustment for age, sex, fasting status (i.e., the interval between consumption of food or drink and blood samples being taken), and a binary variable denoting the genotyping chip used in individuals (the UKBB Axiom array or the UK BiLEVE array) (Po-Ru Loh *et al*, 2015; Tom G Richardson *et al*, 2022).The complete list of summary statistics can be accessed from IEU open GWAS project (https://gwas.mrcieu.ac.uk/ datasets/?gwas_id__icontains=met-d) or MR-Base (https://www.mrbase.org/) website using accessing ID (met-d). After linear regression with adjustment for age, sex, batch as well as the quality control, over 12.3 million SNPs were preserved for downstream analysis.

**Data sources of outcomes**

We used four pancreatitis GWAS summary statistics from FinnGen Round 5 (<https://r5.finngen.fi/>), including acute pancreatitis (3,022 cases and 195,144 controls, ICD-10, K80-K87, https://risteys.finngen.fi/phenocode/PANCREATITIS), alcohol-induced acute pancreatitis (457 cases and 218,335 controls, ICD-10, K85, https://risteys.finngen.fi/phenocode/ALCOPANCACU), chronic pancreatitis (1,737 cases and 195,144 controls, K86.00, K86.01, K86.08, K86.1, https://risteys.finngen.fi/phenocode/K11_CHRONPANC) and alcohol-induced chronic pancreatitis (977 cases and 217,815 controls, ICD-10, K86.00, K86.01, K86.08, https://risteys.finngen.fi/phenocode/ALCOPANCCHRON) for primary analysis. In the secondary analysis, we added two more recently published datasets from UK Biobank: acute pancreatitis (1,748 cases and 454,600 controls, Phecode ID 577.1, ICD-10 K85) and chronic pancreatitis (322 cases and 456,026 controls, PheCode 577.2, ICD-10 K86). The summary statistics can be downloaded from the GWAS catalog with accession numbers GCST90044204 (https://www.ebi.ac.uk/gwas/studies/GCST90044204) and GCST90044205 (https://www.ebi.ac.uk/gwas/studies/GCST90044205).

In FinnGen R5 data, 16,962,023 SNPs were analyzed using SAIGE software based on mixed-model logistic regression (https://github.com/weizhouUMICH/SAIGE/tree/finngen_r5_jk) with adjustments for sex, age, 10 Principal components (PCs) and genotyping batch. For the UK BioBank data, a generalized linear mixed model (GLMM)-based method named (fastGWA-GLMM) was utilized with adjustments for covariates including age, age^2^, sex, age × sex, age^2^ × sex and the top 20 PCs (34737426).

**Analysis with Bayesian model averaging MR (MR-BMA)**

Compared with conventional multivariable MR, the MR-BMA method is particularly useful to investigate high-dimensional datasets (i.e. metabolites) with remarkable genetic correlation (31911605, 33879569). The rationale is that subgroups of metabolites sharing large numbers of genetic variants may act together on the same causal pathway. With MR-BMA, the related metabolites can be disentangled to identify the predominate traits with causal signals.

After univariable MR, we identified the subcategory (fatty acid saturation and triglycerides) showing large numbers of metabolic traits with statistical significance. The traits with a p-value < 0.05 in IVW method were included for MR-BMA. After strict clumping of combined SNPs (R^2^ < 0.001 in 10000 kb distance), we calculated the posterior probability (PP) for each specific model. We used marginal inclusion probability (MIP), which stands for the sum of the PP over all possible models, to rank the traits. Those with the highest MIP rank are suggestive of the strongest “true causal” candidates. Furthermore, the model-averaged causal estimate (MACE), which reflects the average direct effect of each metabolic traits on the outcomes, was computed. Finally, the best models by the PP values (with a PP threshold of 0.02) of the individual models were prioritized. Invalid instruments detected as outliers according to the Q statistics and Cook’s distance were removed and we repeated the aforementioned calculation step to get the best model and estimation values (PP, MIP, causal estimates and MACE)

**Supplementary Figures**

Supplementary figure. 1 Heatmap showing the IVW causal estimates of amino acid in met-d on pancreatitis using two-sample MR. AP, acute pancreatitis; CP, chronic pancreatitis; IVW, inverse-variance weighted; MR, Mendelian randomizations.

Supplementary figure. 2 Heatmap showing the causal estimates of apolipoprotein and cholesterol in lipoprotein in met-d on pancreatitis using two-sample MR. AP, acute pancreatitis; CP, chronic pancreatitis; IVW, inverse-variance weighted; MR, Mendelian randomizations.

Supplementary figure. 3 Heatmap showing the IVW causal estimates of choleseryl ester in met-d on pancreatitis using two-sample MR. AP, acute pancreatitis; CP, chronic pancreatitis; IVW, inverse-variance weighted; MR, Mendelian randomizations.

Supplementary figure. 4 Heatmap showing the IVW causal estimates of free cholesterol in met-d on pancreatitis using two-sample MR. AP, acute pancreatitis; CP, chronic pancreatitis; IVW, inverse-variance weighted; MR, Mendelian randomizations.

Supplementary figure. 5 Heatmap showing the IVW causal estimates of phospholipids in met-d on pancreatitis using two-sample MR. AP, acute pancreatitis; CP, chronic pancreatitis; IVW, inverse-variance weighted; MR, Mendelian randomizations.

Supplementary figure. 6 Heatmap showing the IVW causal estimates of total lipids in met-d on pancreatitis using two-sample MR. AP, acute pancreatitis; CP, chronic pancreatitis; IVW, inverse-variance weighted; MR, Mendelian randomizations.

Supplementary figure. 7 Heatmap showing the IVW causal estimates of other metabolites in met-d on pancreatitis using two-sample MR. AP, acute pancreatitis; CP, chronic pancreatitis; IVW, inverse-variance weighted; MR, Mendelian randomizations.

**Supplementary Tables**

Supplementary Table 1 Heterogeneity of the analyses assessed with Chochrane’s Q, I2, and H statistics in primary analyses.

Supplementary Table 2 pleiotropy of the analyses with MR-Egger pleiotropy test in in primary analyses.

Supplementary Table 3 Association of blood metabolites in met-d dataset with the risk of pancreatitis in primary analyses. MR, Mendelian randomization; IVW, inverse-variance weighted; WM, weighted median

Supplementary Table 4 Effect estimates between genetic variants used as instrumental variables for blood metabolites and the risk of pancreatitis in primary analyses.

Supplementary Table 5 Heterogeneity of the analyses assessed with Chochrane’s Q, I2, and H statistics in secondary validation analyses.

Supplementary Table 6 pleiotropy of the analyses with MR-Egger pleiotropy test in in secondary validation analyses.

Supplementary Table 7 Association of blood metabolites in met-c dataset with the risk of pancreatitis in secondary validation analyses. MR, Mendelian randomization; IVW, inverse-variance weighted; WM, weighted median

Supplementary Table 8 Effect estimates between genetic variants used as instrumental variables for blood metabolites and the risk of pancreatitis in secondary validation analyses.
